# A new method to quantify coronary flow conditions using dynamically scaled in vitro phase contrast magnetic resonance imaging

**DOI:** 10.1186/1532-429X-18-S1-P103

**Published:** 2016-01-27

**Authors:** Susann Beier, John Ormiston, Mark Webster, John Cater, Stuart Norris, Pau Medrano-Gracia, Alistair Young, Brett R Cowan

**Affiliations:** 1 grid.9654.e0000000403723343Anatomy with Radiology, Auckland University, Auckland, New Zealand; 2 Mercy Angiography, Auckland, New Zealand; 3 ADHB, Auckland, New Zealand; 4 Engineering Sciences, Faculty of Engineering, Auckland, New Zealand

## Background

Atherosclerotic coronary artery disease remains a major cause of illness and death, and coronary flow predetermines disease. Limitations in imaging technology prevent coronary flow measurements but computational fluid dynamics (CFD) need sophisticated boundary conditions for accurate flow predictions. MRI has recently been combined with CFD for larger calibre vessels, but small coronary arteries remain inaccessible.

The aim of this study was to assess the feasibility of coronary flow measurement in 3D printed large scale coronary phantoms using phase contrast MRI (PC-MRI).

## Methods

1) Three patient bifurcation geometries with 33°, 72° and 110° angle (mean and ± 2SD of the first principal mode of variation of 300 asymptomatic patients) were 2) 6:1 printed, and their flow was replicated via a dynamically scaled blood mimicking flow circuit. The PC-MRI measured flow was measured was semi-automatically segmented and co-registered to 3) identical, real scale CFD. Measured velocity inlets profiles were transformed and prescribed as CFD inlet condition. The data was statistically compared using a 3D flow field correlation analysis.

## Results

Coronary flow was successfully replicated and measured with dynamically scaled 3D printed phantom PC-MRI, where co-registration (σ<5e-6) resulted in good to strong agreement in magnitude (error 2-12%, ρ ≥0.72), and direction (r^2^≥0.74).

## Conclusions

We have successfully developed, validated and applied a new method to quantify coronary haemodynamics by combining enlarged 3D printed PC-MRI flow with CFD simulations. With this methodology, the PC-MRI measurements can be used to define accurate boundary conditions to elevate CFD simulations and ultimate improve predictions about stent design, coronary artery risk assessment and clinical practice. PC-MRI is non-invasive, accurate imaging technology and has the potential to become an important measurement tool to aid early CFD detection of cardiovascular disease, to risk stratify and optimise treatment for individual patients.

**Figure 1:**
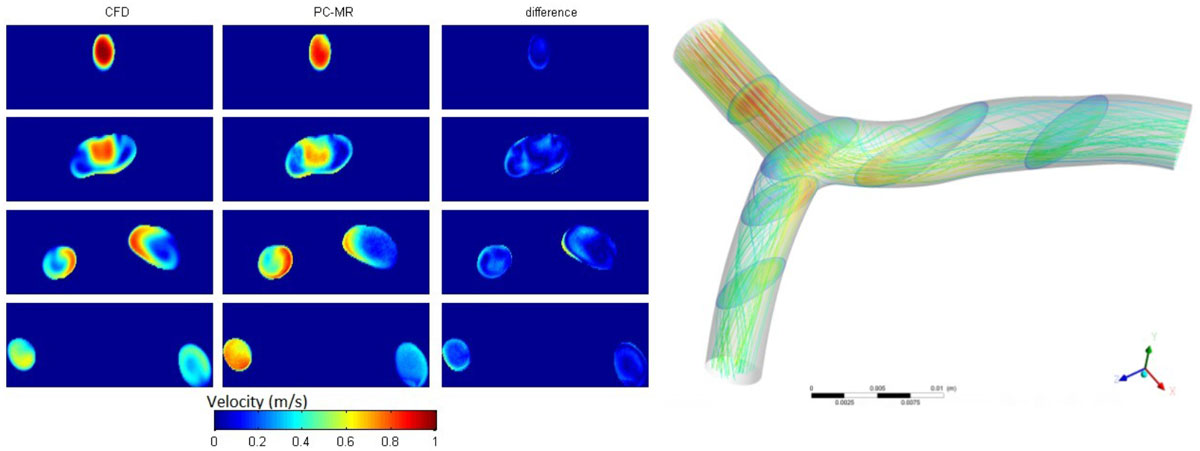
**Detailed flow comparison for the 110 degree patient bifurcation with i) velocity norm contours of real-scale CFD (left), dynamically scalled PC-MRI (middle) and their difference (right) in ii) four planes of the flow field volume**.

